# Identifying Traffic Context Using Driving Stress: A Longitudinal Preliminary Case Study

**DOI:** 10.3390/s19092152

**Published:** 2019-05-09

**Authors:** Olga Vl. Bitkina, Jungyoon Kim, Jangwoon Park, Jaehyun Park, Hyun K. Kim

**Affiliations:** 1 Department of Industrial and Management Engineering, Incheon National University (INU), Incheon 22012, Korea; olgabitkina@inu.ac.kr; 2 Department of Computer Science, Kent State University, Kent, OH 44242, USA; jkim78@kent.edu; 3 Department of Engineering, Texas A&M University—Corpus Christi, Corpus Christi, TX 78412, USA; Jangwoon.Park@tamucc.edu; 4 School of Information Convergence, Kwangwoon University, Seoul 01897, Korea; hyunkkim@kw.ac.kr

**Keywords:** artificial intelligence, driving stress, electrodermal activity, road traffic, road types

## Abstract

Many previous studies have identified that physiological responses of a driver are significantly associated with driving stress. However, research is limited to identifying the effects of traffic conditions (low vs. high traffic) and road types (highway vs. city) on driving stress. The objective of this study is to quantify the relationship between driving stress and traffic conditions, and driving stress and road types, respectively. In this study, electrodermal activity (EDA) signals for a male driver were collected in real road driving conditions for 60 min a day for 21 days. To classify the levels of driving stress (low vs. high), two separate models were developed by incorporating the statistical features of the EDA signals, one for traffic conditions and the other for road types. Both models were based on the application of EDA features with the logistic regression analysis. City driving turned out to be more stressful than highway driving. Traffic conditions, defined as traffic jam also significantly affected the stress level of the driver, when using the criteria of the vehicle speed of 40 km/h and standard deviation of the speed of 20 km/h. Relevance to industry: The classification results of the two models indicate that the traffic conditions and the road types are important features for driving stress and its related applications.

## 1. Introduction

Previous studies have identified that the level of driving stress could be affected by different driving conditions [1,2,3,4], such as types of roads [5,6], traffic congestion [7], and weather [8]. Dwight and David [7] identified the relationship between traffic conditions and stress levels based on driver interviews. They found that stress was higher for drivers who have experienced traffic congestions. Therefore, aggressive driving behaviors were observed more in high congestion areas than lower ones. Hill and Boyle [8] studied how different driving tasks and roadway conditions influence the stress perceived by drivers. They conducted a survey to assess drivers’ stress under various roads, traffic conditions, and weather-related scenarios. The results of this study showed that driving stress was influenced by not only driver characteristics (age, gender, etc.) but also landscape types and driving distances. Therefore, we can assume that road traffic conditions and road types are associated with driving stress.

One of the most accurate indicators of driver stress is the electrodermal activity (EDA) [9,10,11,12], which characterizes the activity of electricity on human skin due to sweat [13]. Zangroniz et al. [14] found that the EDA signal is an accurate measure to distinguish calm/stressful conditions. Healey and Picard [5] presented methods for collecting and analyzing EDA data to detect driver stress during various driving conditions on actual roads. Healey and Picard found that EDA and heart rate metrics are the most significantly correlated with driver stress. Rigas et al. [15] presented a novel methodology, based on a dynamic Bayesian network for the estimation of driver stress in specific driving events, using an electrocardiogram (ECG) and EDA signals. Singh et al. [16] studied the feature extraction method and a few algorithms for detecting stress using EDA, ECG, and photoplethysmography (PPG) signals. Munla et al. [17] used heart rate variability analysis for driving stress detection. Lal and Craig [18] studied the psychophysiological changes that occurred during a driver simulator task based on biological signals.

There has been a series of machine learning algorithms that can be applied to detect different events in various research fields by using physiological signal analysis. Plawiak et al. [19,20] applied deep genetic ensemble of classifiers to detect arrhythmia using the ECG signal and artificial neural network to estimate the state of consumption of a pump, based on dynamic pressure and vibrations. Ksiazec et al. [21] used a machine learning approach to detect Hepatocellular Carcinoma using physiological features. Rzecki et al. [22,23] proposed the computational intelligence methods for person recognition using biometric features and used the same method for the automated identification of paper-ink samples through laser-induced breakdown spectroscopy.

Also, many studies have identified that traffic conditions and road types are associated with the levels of driving stress. For the studies of the relationship between traffic conditions and driving stress, Dwight and David [24] reported that a driver’s psychological state depends on the road traffic situation, and driving stress is greater in high congestion areas than in low congestion areas. Neighbors et al. [25] identified that slow traffic was linked to greater feelings of pressure and stress. Meanwhile, traffic congestion occurs when the traffic density is exceeded due to a large number of vehicles. According to the traffic flow theory [26], there are a few important traffic flow parameters, namely speed of vehicles, flow (vehicles per hour), density (number of vehicles occupying a given length of highway or lane), and road capacity. In turn, some studies [27,28] suggest that two of the many important characteristics of road traffic are average speed and standard deviation. Table 1 shows that previous research proved the viability of vehicle speed as a characteristic of traffic congestion.

**Figure 1:**
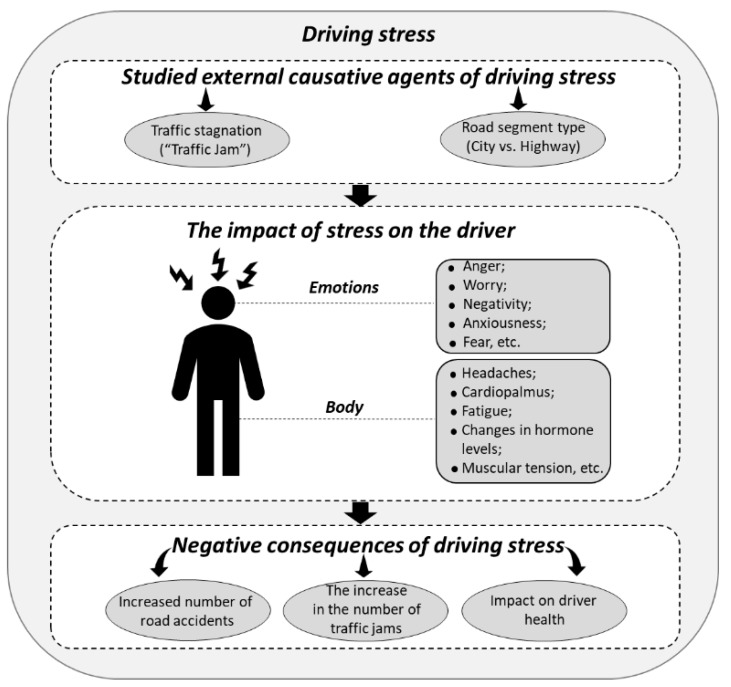
Relationship between driving stress and studied factors.

Based on available research, the current study shows a simplified hypothesis that considers only two traffic congestion parameters, namely mean speed (MS) and standard deviation of speed (STDS). The stress level of the driver was assumed to be high in high traffic conditions and low in low traffic conditions. For the studies of the relationship between road types and driving stress, Liu and Du [10] detected low, medium, and high stress levels using an EDA signal and they found that these levels corresponded to no-driving, highway driving, and city driving conditions, respectively. According to Healey and Picard [5] and Westerink et al. [6], city driving is more stressful than highway driving. Based on the previous studies, we can assume that low and high levels of stress correspond to highway and city driving, respectively. Figure 1 shows an overview of the studied factors including traffic conditions as well as road types in this study, their impact on driving stress in terms of mental and physical, and their consequences, such as increasing risks of accidents and decreasing driving performance.

Summarizing the above information, Table 2 shows a brief compilation of studies which reflects the previous research trends in driving stress detection.

The distinctive features of our paper compared with previous research are following conditions and their combinations. The experimental route included city and highway roads without a driver rest time. The driver was influenced by different road types and unstable traffic conditions at the same time. EDA was used as a driving stress measure and for its analysis the special signal features were extracted. Based on Table 1 the vehicle speed signal was used as a traffic conditions indicator and new traffic congestion parameter was extracted by authors. Although many previous studies have identified the relationship between driving stress and traffic conditions, and driving stress and road types, our best knowledge has developed the classification models of driving stress by considering the traffic conditions and road types. In the previous study, most focus has been on the driver’s emotional state or stress state. This study was motivated to investigate the relationship between driver stress conditions, road conditions, and road type. We tried to define and predict the traffic jam state itself considering the driver’s bio-signals, in addition to the analysis of the road type. The method in this study is expected to contribute to defining a traffic jam. To do this, information was collected on actual roads for a month.

## 2. Methods

### 2.1. Participant

The participant of the experiment was a healthy 35-year-old Korean male with a height of 176 cm and a weight of 67 kg. The driver was well acquainted with the driving route and had driving experience of more than 15 years.

### 2.2. Apparatus

The Empatica E4 wristband (EDA sensor) was used for physiological signal collection in our experiment. The wristband [42] is a wearable and wireless device designed for comfortable, continuous, and real-time data acquisition in daily life. It contains three sensors: (1) PPG sensor that detects cardiovascular features such as blood volume pulse and heart rate variability, (2) EDA sensor that measures an arousal of a sympathetic nervous system and derive features related to stress, engagement, and excitement, and (3) three-axis accelerometer that captures motion-based activity. The wristband operates either in a streaming mode for real-time data viewing on a mobile device, using Bluetooth Low Energy or in recording mode, using its internal memory. In our study, EDA data were collected using the recording mode. The EDA sensor has the following characteristics: sampling frequency of 4 Hz, resolution of one digit –900 pSiemens, range of 0.01–100 μSiemens, and alternating current (8 Hz frequency) with a maximum peak to a peak value of 100 μAmps (at 100 μSiemens) [37].

To record the vehicle movements and on-road situations, a standard on-board diagnostic system (OBD-II) was used. OBD-II is a computer-based system built into modern passenger cars, which monitors emission-related controls, and performance of the engine and also detects malfunctions. OBD-II systems provide access to the health information of a vehicle along with numerous parameters and sensors from the engine control unit (ECU). The OBD-II system offers valuable information, including diagnostic trouble codes, when troubleshooting problems [43]. The technical characteristics of OBD-II can be found in the standard signaling protocols for interfaces.

A mid-sized sedan, Hyundai Grandeur (Azera in the U.S.) 6th generation (Hybrid), was used in this study. The humidity values inside the vehicle were maintained between 1016 and 1030 Mbar throughout the experiment.

### 2.3. Experimental Conditions

**Figure 2:**
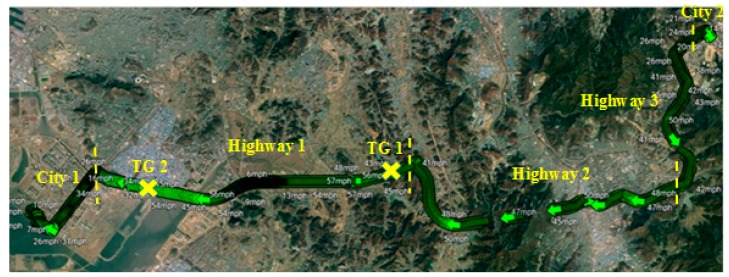
Experiment on the driving route between Incheon and Seoul (captured by Google Earth).

In this study, an experiment was conducted during real vehicle driving between the cities of Incheon and Seoul in South Korea, which is a fifty-kilometer route consisting of five main segments: City 1 (Incheon in Korea), Highway 1, Highway 2, Highway 3, and City 2 (Seoul in Korea) with two Tollgate points (TG) (Figure 2). The total time for a one-way drive was approximately 60 min. During this route, two types of recorded data were collected, such as EDA data measured by the Empatica E4 sensor (sampling rate = 4 Hz) and speed signal measured by OBD-II.

All studied data were collected for a month from 13 December 2017 to 13 January 2018. Datasets for 25 weekdays were obtained. Data from four days were omitted because they were inaccurate and unclear, such as unrecorded time data, device failure and zero values of EDA data. The total number of datasets (days) used in the analysis was 21.

### 2.4. Measures

**Figure 3:**
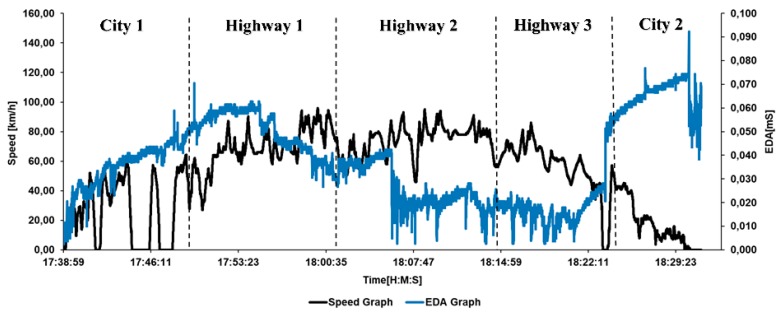
Line plot of the collected speed and electrodermal activity (EDA) data in the same time series for an evening session.

Two methods of driving stress evaluation were studied and compared: Dependency between stress and road traffic, and between stress and road type. The vehicle speed and the driver’s EDA signals were recorded for different road segments such as city and highway driving as shown in Figure 3.

The road traffic conditions were classified as a low traffic or high traffic state based on the vehicle speed. We determined the high traffic criteria set in Table 3, which characterize potential traffic congestion. The occurrence of high traffic corresponds to high driving stress and its absence to low driving stress. Average speed and standard speed deviation were calculated for every six-minute window of speed data. Speed data for every six-minute window were automatically checked and compared with the adjusted average speed and standard speed deviation sets in Table 3 to determine the potential traffic congestion. If an average and standard deviation of the vehicle speed during a certain period were lower than the criteria set (Table 3), then that period was classified as high traffic conditions, and the other period was classified as low traffic conditions. The driver’s stress level was assumed to be low in low traffic conditions and high in high traffic conditions.

For the second model of dependency between road type and stress state, it was considered that city driving results in a high stress level, and highway driving results in a low stress level. We determined that city driving causes higher stress due to a large number of pedestrians, traffic lights, and traffic congestion. In contrast, such difficult driving conditions are less likely to occur when driving on highways. Therefore, highway driving was classified as low stress driving.

We analyzed the extracted EDA features for each road segment, with a driving time of about 35–40 min on highway-type segments, and 20–25 min on city segments, for a total duration of approximately 60 min. The segments with overlapped periods of high and low levels of stress were excluded. Feature extraction is an important signal processing step for finding more dominating information and for reducing the volume of auxiliary research procedures. It is known that, depending on the type of the signal, different features can be extracted. In this study, for physiological EDA signals, the features proposed by Healey and Picard [5], amplitude (OM) and duration (OD) calculated from signal peaks and valleys, were extracted.

**Figure 4:**
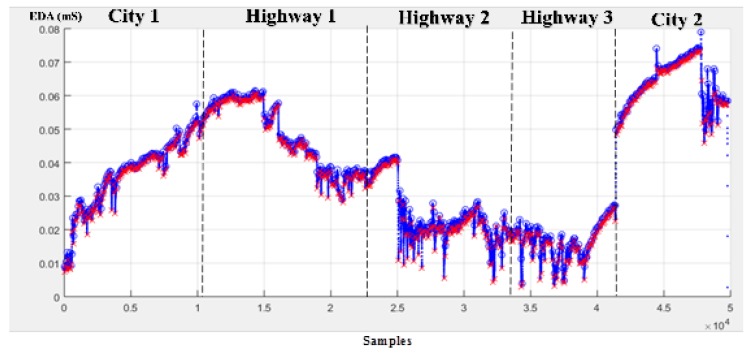
EDA signal processing for January 11, 2018 morning session (peaks and valleys are marked by ○ and ×, respectively).

In the current study, the processed EDA signal was resampled at 15.5 Hz. Based on the calculation of mean OD ± 3 × standard deviation (SDOD), it was found that 99.7% of OD falls within the confidence interval. This means that the six-minute window size (approximately equal to 5580 samples) meets the accuracy requirements. In this study, a one-minute sliding window was determined, which was approximately equal to 930 samples, and the moving average window was 60 samples. To find the signal peaks and valleys, the “findpeaks” function in MATLAB (R2017 version) was used. The signal feature extraction process allows us to extract the following EDA characteristics: minimum (min OD and min OM), maximum (max OD and max OM), mean (mean OD and mean OM), standard deviation (SD OD and SD OM), summation (sum of ODs and sum of OMs), and the number of occurrences of duration and amplitude (nOD and nOM). Figure 4 shows the results of applying the OM and OD extraction algorithm for the morning driving session on 11 January 2018.

### 2.5. Analysis Method

**Figure 5:**
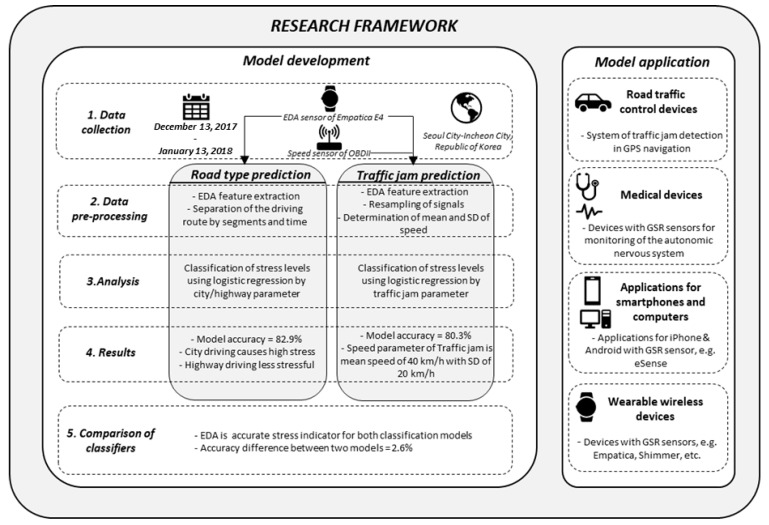
Stepwise development and application of the models.

Logistic regression analyses were performed, by using IBM SPSS Statistics Version 23 Software, on every traffic conditions and different road types. Figure 5 contains the schematic description of the development process for both models and their application areas. The physiological features of EDA (OD and OM) and OBD-II data (vehicle speed) were used to construct the same framework in this study. In particular, the model development section of Figure 5 summarizes the study in detail, which is designed to identify the abstract content shown in Figure 1.

The development process in Figure 5 was performed in five steps: Data collection, data pre-processing, analysis, results, and comparison of classifiers. The data collection step shows the period, place, and used sensors during the experiment. Data pre-processing introduces the preliminary processing steps on obtained data for each method. Analysis and results show the analytical methods used, and the main results obtained. Comparison of classifiers provides general comparison for both methods. The model application describes the most applicable areas for the developed methods, such as road traffic management, medicine, and electronic devices.

Based on previous studies in the introductory section, key parameters of physiological signals, road types and traffic situation characteristics have been selected as classifiers. The most important EDA features related to driving stress are minimum, maximum, mean, standard deviation, sum, and the number of occurrences of OD and OM. The most important driving conditions affect the mental state of the driver. Road type [5,6] and traffic jam [24] are representative elements. Based on this, the current study classified the road types and identified traffic jams using the vehicle’s speed and the standard deviation of the speed [27,28]. A summary of the models used in this study is shown in Table 4.

For a complete evaluation of the classification model efficiency, the accuracy (A), sensitivity (Sn), specificity (Sp), and positive predictive value (PPV) were calculated additionally as the specifying characteristics (see Table 3):

A = (cases of high stress + cases of low stress)/(all cases of stress) = TP + TN/(TP + TN + FP + FN),(1)

Sn = (cases of high stress)/(all cases of stress) = TP/(TP + FN),(2)

Sp = (cases of low stress)/(all cases of low stress) = TN/(TN + FP),(3)

PPV = (cases of high stress)/(all cases of high stress) = TP/(TP+FP),(4)

In Equations (1)–(4), FP is the false positive, FN is the false negative, TP is the true positive, and TN is the true negative data. The accuracy determines the ratio of correct predictions to the total analyzed cases. False positives and false negatives are cases when the developed classifier erroneously recognizes low stress as high stress and high stress as low stress, respectively. True positives and true negatives are cases when the developed classifier recognizes stress levels correctly.

## 3. Results

### 3.1. Traffic Conditions

A combination of 40 km/h of average speed and 20 km/h of standard deviation, which exhibit relatively higher accuracy, sensitivity, specificity, and positive predictive value, was selected. The mean values of four measures at the combination were the highest. It is highlighted in bold in Table 3. From Table 3, we can see that a few cases have an accuracy of over 80%. However, we cannot select them due to low sensitivity and predictive value, which are under 50%. Low sensitivity shows that a large number of cases with high stress are not recognized, and the low predictive value indicates the low effectiveness of the model in detecting high stress. Thus, considering all the effective parameters of the developed model, the most accurate criterion of low vs. high traffic conditions was found to be with average speed of 40 km/h and standard deviation of 20 km/h: Classification model accuracy = 80.3%, sensitivity = 85%, specificity = 78%, and positive predictivity = 70%. Based on these results, we can conclude that traffic conditions (low vs. high traffic) are an important element to classify driving stress levels (low vs. high stress). In addition, we presumed that traffic conditions (average speed < 40 km/h and SD < 20 km/h) could be a clear threshold of driving stress levels (low vs. high stress), compared to the other 19 conditions in Table 3.

Table 5 shows the developed model for the classification of low stress and high stress levels depending on the high traffic. In the traffic condition model, only NOM was used because the variables NOD and NOM are the same.

It was found that three extracted features (number of occurrences (N), mean amplitude (MeanOM), and maximal amplitude (MaxOM)) of the hand EDA data are significant (all ps < 0.05). The mathematical rule of the developed model for the classification of stress levels depends on the probability concept. High stress was recognized by the model if the probability of case i was greater than 0.5. Otherwise the case was classified as low stress.

**Figure 6:**
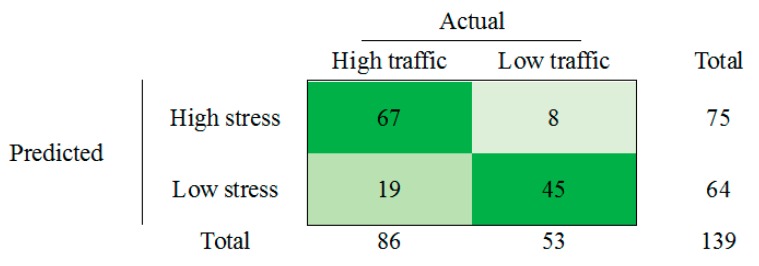
Confusion matrix of the model using the traffic condition datasets.

The goodness of model fit measure evaluates the ability of developed models to explain variation in the dependent variable (high and low stress). As such measures, pseudo-R-squared are usually used in logistic regression analysis. In the presented study pseudo-R-squared of Cox and Snell and Nagelkerke were obtained using IBM SPSS Statistics Software (Version 23). For traffic condition model Cox and Snell and Nagelkerke R-squares are 0.323 and 0.432 respectively. Figure 6 shows the confusion matrix of the analyzed datasets for the classification of high and low stress levels by using the EDA data, depending on the traffic conditions.

### 3.2. Road Type

In the second method for road type prediction, where driving in cities was recognized as a high stress state and highway as a low stress state, the statistical method accuracy reached 82.9% with sensitivity of 81%, specificity of 84%, and positive predictivity of 65%. Table 6 shows the developed model including all used predictors for road type segments.

**Figure 7:**
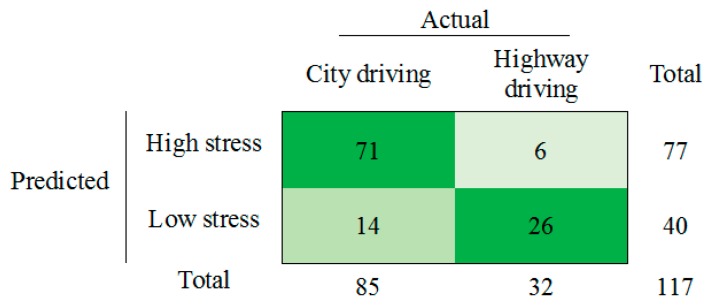
Confusion matrix of the model using the road type datasets.

The binary logistic regression model for road type segments shows that Min OD is significant (*p* < 0.05). For road type model goodness of fit was presented by Cox and Snell and Nagelkerke R-square with values of 0.374 and 0.518, respectively. As for the traffic condition model, pseudo-R-squared was obtained through SPSS Statistics Software (Version 23). Figure 7 shows the confusion matrix of the model developed by the road types.

## 4. Discussion

### 4.1. Predictability of Stress State Depending on Traffic Conditions and Road Type

External driving conditions have a decisive influence on the emotional state of the driver and the occurrence of traffic accidents [44,45]. Based on this, two driving conditions of traffic congestion and road type were chosen and compared in this study. Simplified traffic conditions criteria, which characterize traffic congestion, were found to be 40 km/h of average speed with a standard deviation of 20 km/h. If this criterion is met, then we can say that it corresponds to potential traffic congestion and in this moment, the driver is in a high stress state. To confirm this theory, a statistical model was developed. It shows the classification accuracy of 80.3%. From the detected EDA features, the number of occurrences, duration, and amplitude of peaks and valleys were found as significant predictors for the analyzed classification model. The method for determining stress dependency on the road type segments shows the classification accuracy of 82.9% with a significant predictor of Min OD. The goodness of model fit is assessed using various measures and in modern literature there is no consensus which one is better [46]. In our study it was evaluated using Cox and Snell and Nagelkerke pseudo-R-squares, which show improvement of the constant-only model (null model) after applying all predictors (full model with predictors). Cox and Snell pseudo-R^2^ is not able to reach “1” even for the perfect model, in turn Nagelkerke R^2^ is adjusted Cox and Snell R^2^ and its maximal value can be extended to “1” [46]. Obtained results for traffic condition model show that model explain between 32.3% (Cox and Snell) and 43.2% (Nagelkerke) of variance in low and high stress level occurrence, in turn, road type model explain between 37.4% (Cox and Snell) and 51.8% (Nagelkerke) of variance (Table 7). In previous studies, there is no consensus on how to interpret the values of pseudo-R-squares, but some sources [47,48] evaluated the Cox & Snell level higher than 0.3 and Nagelkerke level higher than 0.35 as a satisfactory. Based on this, both our models have a good level of compliance with the observations, but the road type model has higher values and it fits better the observations. The comparison of both methods is shown in Table 7. We also compared the performance of other classifiers using 10-fold cross-validation and separation of training (70%) and testing (30%) data. As shown in Table 8, overall, random forest (RF) has the best performance, the area under the ROC curve (AUC) values of RFs are 85.70% and 79.10% in 10-fold cross-validation and 89.46% and 85.82% in training/testing method, respectively. Surprisingly, multi-layered perceptron which is the neural network-based method shows the overall low performance. Thus, the performance of the tree-based methods is better than neural network-based method in this type of data.

Sensitivity and specificity each reflect how well the model developer is able to grasp high stress and low stress, respectively. A positive predictive value indicates the exactness of high stress. Experimental results show that the overall model of the road type predominates, but it can predict the stress more easily in the traffic-condition-based model. We use the AUC value which reflects the overall performance better as a single value.

Initial hypothesis about dependency between driving stress, traffic congestion, and road type segments was confirmed. Both methods show accuracies higher than 80% with a small difference of 2–3%, which means that these models are effective and can be used for stress prediction in real driving conditions. Certainly not only these factors have an impact on the predictability of a driving stress state, for example, the internal negative emotions and experiences may affect the driver’s state [24,49]. The distinction between these two groups of factors is a difficult scientific task. Based on this, one of the future scientific questions is the improvement of stress detection methods, considering the personal characteristics and emotions of the drivers.

Next factor that can additionally affect the predictability of the stress state is stress measures. Villarejo et al. [50] reported that EDA successfully detects various human emotional states, but it is difficult to distinguish between, for example, the stress situation and the situation of making an effort by a human. EDA sensor may recognize these two different states as the same. Additionally, previous studies devoted to the stress recognition proposed various measures, such as heart rate, blood pressure, muscle tension, etc. [51,52]. In our paper, EDA signal was used as a significant measure of the stress level. It was shown that along with the mentioned characteristics from previous studies, EDA successfully can be used to predict low/high stress during driving in real time. Moreover, EDA sensor is more convenient and useful for long-term monitoring since this type of sensors does not always require the gel-type electrodes and specific contact points on human skin unlike EEG and ECG sensors.

In the presented study, classical statistical models based on logistic regression analysis were developed. In turn, previous studies show that different approaches can be used for stress and emotional state recognition. Rigas et al. [53] proposed a model to predict driving stress (high, medium and low) using EDA and ECG signals based on a dynamic Bayesian network with an accuracy range of 31–94%. Magana et al. [54] introduced the deep learning algorithms based on the heart rate variability (HRV) signal to estimate the stress of drivers and passengers with an accuracy range of 86–92%. Jabon et al. [55] used a few classifiers, including Bayesian nets, decision tables, decision trees, support vector machines, regressions, and LogitBoost to predict unsafe driver behavior by a facial expression based on Kappa values (in most cases, LogitBoost classifier provided the highest Kappa statistic). By comparing these with the methods proposed in our paper, it should be noted that elements of advanced deep learning can be considered in the future to increase the prediction ability of the developed models. From the viewpoint of average accuracy, the presented models show satisfactory results. The proposed models can be extended to include more variables or features, but for other classifiers, there can be limitations due to algorithm inflexibility. Using presented models needs minimal efforts and simple devices. Additionally, it can be simply applied for science and industry. Otherwise, some advanced approaches can be more difficult. In general, the combination of developed and more advanced classifiers can result in offering applications with improved accuracy and predictability.

Our study demonstrates and compares two developed models capable of a satisfactory accuracy to predict the stress state of the drivers based on physiological signals. Obtained results expand previous research and new findings can be used in traffic management, medicine, and design of electronic devices with physiological sensors.

### 4.2. Limitation of This Study and Future Research

The presented research is the initial result of a long-term study about stress prediction based on physiological signals. Even though both developed classification models showed good results, there are a few limitations, which will need to be addressed in the future. First, a shortcoming of this study is the number of participants, which was limited to one male driver. The authors compensated for it by increasing the experimental period to one month to obtain the optimal number of driving datasets for analysis. Second, the temperature in the car was not regulated during the experiment. It will need to be controlled in future studies because this condition can possibly affect the psychological state and sweating intensity of the driver. Third, the traffic congestion concept is a simplified point of view. The concept of the traffic flow theory [26] will be used in future research. From this theory, the speed of vehicles, traffic flow, density, road capacity, and distance between vehicles will be considered to extend the developed methods. Fourth, the accuracies of models obtained can be improved by increasing their sensitivity to different emotional states of the driver, such as anger, annoyance, stress, etc., as discussed in the previous studies [24,49,50].

Few additional observations will need to be considered in the future. For example, direct EDA data evaluation shows that average EDA data are approximately doubled in the morning route (EDA = 0.03129 mS) than in the evening route (EDA = 0.01543 mS). A higher EDA level was observed in the morning session on 12 January 2018, which was the coldest observed day with a temperature of −14 °C in the experimental period. Potentially, this result indicates that weather conditions and the time of day affect the driver’s EDA signal, and these factors will be considered in the future. This and further research can find many applications, including the development of wearable devices containing physiological sensors.

## 5. Conclusions

This study presented the findings on the levels of driving stress depending on the traffic conditions and road types, using the EDA signals. The study confirmed that EDA is a significant indicator of the psychological stress and an effective tool to determine the stress levels in real driving conditions. Advantages of the developed method are that both developed models have an ability to predict stress levels under actual driving conditions with over 80% accuracy. The developed models can also be used to classify driving stress in different traffic situations and different road types. The definition of a traffic jam state in a driving situation is one of the important contributions of this study. One of the characteristics of the models is the ability to classify the stress level without considering the rest state of the driver. This makes the models universal for use in a variety of situations without the preliminary intervention. The proposed model and the experimental procedure are expected to be easily reconfigured by other researchers. One of the disadvantages of the proposed model is that the traffic congestion concept needs to be defined and improved more specifically based on traffic flow theory. To make a better model in the future, the following points should be considered. First, researchers can collect more road information, such as lane-keeping status, as well as bio-signals, such as ECG. In addition, various kinds of machine-learning techniques can be applied instead of logistic regression. Finally, researchers can incorporate other factors, such as seasonal factors, in-vehicle temperature, and a driver’s attention into the model as key features.

Obtained results can be used for practical application. Examples of such uses are sensors for monitoring of the autonomic nervous system, smartphone/computer applications and wearable wireless devices with EDA sensors.

## Figures and Tables

**Table 1:** Speed as an indicator of traffic congestion [24,25,26,27,28].

References	Brief Description
Pattara-Aticom et al. [29]	Authors classified three levels of traffic congestion based on GPS speed data using threshold technique. It was shown that vehicle velocity is an important characteristic of traffic congestion.
Palubinskas et al. [30]	Authors introduced the traffic congestion detection approach for image time series and found that average velocity is main the traffic parameter.
Thianniwet et al. [31]	Authors proposed a technique to identify road traffic congestion levels using velocity data from a GPS device. Vehicle moving pattern as an important element was extracted through the sliding window technique.
Xing et al. [32]	Authors studied the road tunnel traffic safety and built up the traffic assessment model contained the parameter of speed variance. It was shown that speed variance is an important element of traffic evaluation.
He et al. [33]	Authors analyzed traffic congestion in urban road networks using speed data. The speed performance index was found as the indicator of road state for congested or smooth traffic.

**Table 2:** Previous studies on driving stress.

References	Collected Mental and Physical Data	Studied Factors	Analysis Methods
Xing et al. [34]	ECG, eye movement, flicker value, face image, self-reported emotional state	Road conditions (three different highways), traffic conditions, driving environment, vehicle behavior	Questionnaire, detection and processing of low/ high-frequency ratio of heart rate variability
Matthews et al. [35]	Self-reported emotional state	Age, type of road (city road, intercity road), frequency of car use, driving conditions (pre-drive, post-drive, weekend), accident involvement, speeding convictions	Questionnaire, factor analysis, ANOVA
Singh et al. [32]	GSR, PPG	Urban driving scenarios (pre-driving, relax driving, busy driving, return driving, rost-driving)	Detection and processing the GSR/PPG signals
Keshan et al. [36]	ECG	Type of road (city road, highway)	Detection and processing the ECG signal
Goel et al. [37]	ECG	Real-time driving in normal road conditions	Detection and processing the ECG signal
Riener [38]	ECG, self-reported emotional state	Specific route, fixed daytime	Post-experiment interview, Detection and processing of low/ high frequency ratio of heart rate variability
Lee et al. [39]	ECG, PPG	Real-time driving in a busy narrow street	Detection and processing the ECG signal
Mundell et al. [40]	GSR	Alternation of rest and driving periods	Detection and processing the GSR signal
Kurniawan et al. [41]	Speech signal, GSR	Real-time driving in usual road conditions	Detection and processing the Speech and GSR signals

**Table 3:** Models with various averages and standard deviations of vehicle speed.

Average Speed (km/h)	Standard Deviation (km/h)	Accuracy (%)	Sensitivity (%)	Specificity (%)	Predictive Value (%)
20	10	77.3	78	77	64
	15	78.7	68	81	40
	20	85.8	50	87	15
	25	92.9	0	93	0
	30	97.2	0	97	0
30	10	70.9	74	68	67
	15	78	81	76	64
	20	73	67	75	38
	25	87.1	82	88	36
	30	88.6	80	89	36
**40**	10	71.6	73	69	75
	15	72.3	75	71	65
	**20**	**80.3**	**85**	**78**	**70**
	25	76.6	70	78	43
	30	79.4	72	81	41
50	10	75.9	79	71	79
	15	70.9	73	68	74
	20	73.8	76	72	66
	25	79.4	82	78	68
	30	75.9	70	78	53

**Table 4:** Specification of classification models.

Classification Model	EDA Signal Features	Driving Conditions Features	Analytical Method	Accuracy
Road type prediction	amplitude and duration (min, max, mean, SD, sum, N)	Separation of city and highway section of the path	Logistic regression	82.9%
Traffic jam prediction	amplitude and duration (min, max, mean, SD, sum, N)	Determination of traffic jam criteria using vehicle speed and speed SD	Logistic regression	80.3%

**Table 5:** Model based on traffic conditions (low vs. high traffic).

Predictor	Coefficient	*p*-Value
N	−0.117	0.046
Mean OM	−657.549	0.040
Max OM	66.019	0.047
Min OM	1586.879	0.075
Sum OM	7.747	0.063
SD OM	71.514	0.678
Max OD	0.001	0.727
Min OD	−0.005	0.219
Sum OD	0.000	0.487
Mean OD	−0.001	0.941
Constant	5.444	0.198

**Table 6:** Model based on different types of road segments.

Predictor	Coefficient	*p*-Value
Min OD	0.011	0.031
Max OD	0.000	0.977
Sum OD	0.000	0.378
Mean OD	−0.009	0.240
Mean OM	−128.868	0.604
Max OM	−1.864	0.918
Min OM	18.381	0.976
Sum OM	4.682	0.176
SD OM	94.897	0.383
N	−0.062	0.145
Constant	3.740	0.218

**Table 7:** Comparison of two developed methods.

Method	A (%)	Sn (%)	Sp (%)	PPV (%)	Cox & Snell R^2^	Nagelkerke R^2^
Traffic conditions	80.3	85	78	70	0.323	0.432
Road Type	82.9	81	84	65	0.374	0.518

**Table:** (a) 10-fold cross-validation.

10-Fold Cross-Validation	ROAD TYPE				Traffic Condition			
	Sn	Sp	PPV	AUC	Sn	Sp	PPV	AUC
RF	**64.70**	**88.20**	**76.70**	**85.70**	**60.90**	**86.70**	**79.60**	**79.10**
AB	62.70	90.60	80.00	**86.10**	57.80	88.00	80.40	68.90
NB	52.90	95.30	87.10	84.70	53.10	86.70	77.30	75.60
SVM	56.90	89.40	76.30	73.10	70.30	65.30	63.40	67.80
MLP	54.90	83.50	66.70	75.50	57.80	80.00	71.20	73.80

**Table:** (b) Training 70% and testing 30%.

Testing (30%) Training (70%)	Road Type				Traffic Condition			
	Sn	Sp	PPV	AUC	Sn	Sp	PPV	AUC
RF	**76.47**	**87.50**	**81.25**	**89.46**	**75.00**	**92.31**	**85.71**	**85.82**
AB	76.47	83.33	76.47	83.46	68.75	57.69	50.00	62.74
NB	47.06	91.67	80.00	83.09	68.75	61.54	52.38	73.80
SVM	52.90	91.70	81.80	72.30	12.50	100.00	100.00	59.62
MLP	52.94	62.50	50.00	64.22	52.20	57.90	60.00	54.33

* Random forest (RF), adaBoost (AB), naïve Bayes (NB), support vector machine (SVM), multi-layered perceptron (MLP).
